# Improving risk assessments for CAMHS admissions at Great Ormond Street Hospital

**DOI:** 10.1192/bjo.2021.316

**Published:** 2021-06-18

**Authors:** Lisanne Stock, Sacha Evans

**Affiliations:** Great Ormond Street Hospital

## Abstract

**Aims:**

During the COVID-19 pandemic, admissions to the Mildred Creek Unit (MCU), an Inpatient CAMHS Ward at Great Ormond Street Hospital (GOSH) changed.

The MCU is a 7–10 bed unit for children aged 7–15 years based on therapeutic milieu principles. The ward accepts patients via a planned national referral pathway, however, during the COVID-19 pandemic, patients were admitted as emergencies and consequently risk assessments were missed. Risk assessment is important in all admissions and as the MCU is not a locked unit, early risk assessment is particularly important.

We aimed to review whether risk assessment occurred within one working day of admission, as suggested by the ward risk assessment policy, and if this was not the case, our aim was to ensure that all risk assessments took place within this period via our audit interventions.

**Method:**

We collated data looking at the time between admission to GOSH and the date at which first risk assessments took place. We then put in place three interventions.

1. Posters prompting doctors who were providing on-call liaison input to perform a risk assessment within one working day of admission.
2. New junior doctors were provided with written and verbal information to emphasise the importance of early risk assessment.
3. Guidelines also highlight that assessment of risk may need to be on-going. We therefore added a prompt section in the weekly ward round proforma with the aim of reducing the interval between risk assessments during admission.

The first audit cycle was conducted on the 3/8/2020 and the second on the 28/11/21 to allow for a comparative number of inpatients between the first and second audit cycle.

**Result:**

We found these interventions significantly reduced delays in risk assessments. Prior to the audit's first cycle the average delay between admission to GOSH/MCU and a risk assessment was 2 weeks. After the interventions there were no patients whose risk assessment was delayed outside the next working day parameters.

**Conclusion:**

This full cycle audit demonstrates the impact that prompts to clinical practice can make on patient care. It is important to recognise the need for flexible risk assessment with regular review, especially at times of clinical change. We hope that this continued trend for early risk assessment leads to improved clinical care and timely discussion of risk for all new CAMHS inpatients at GOSH.

